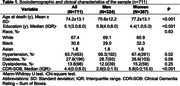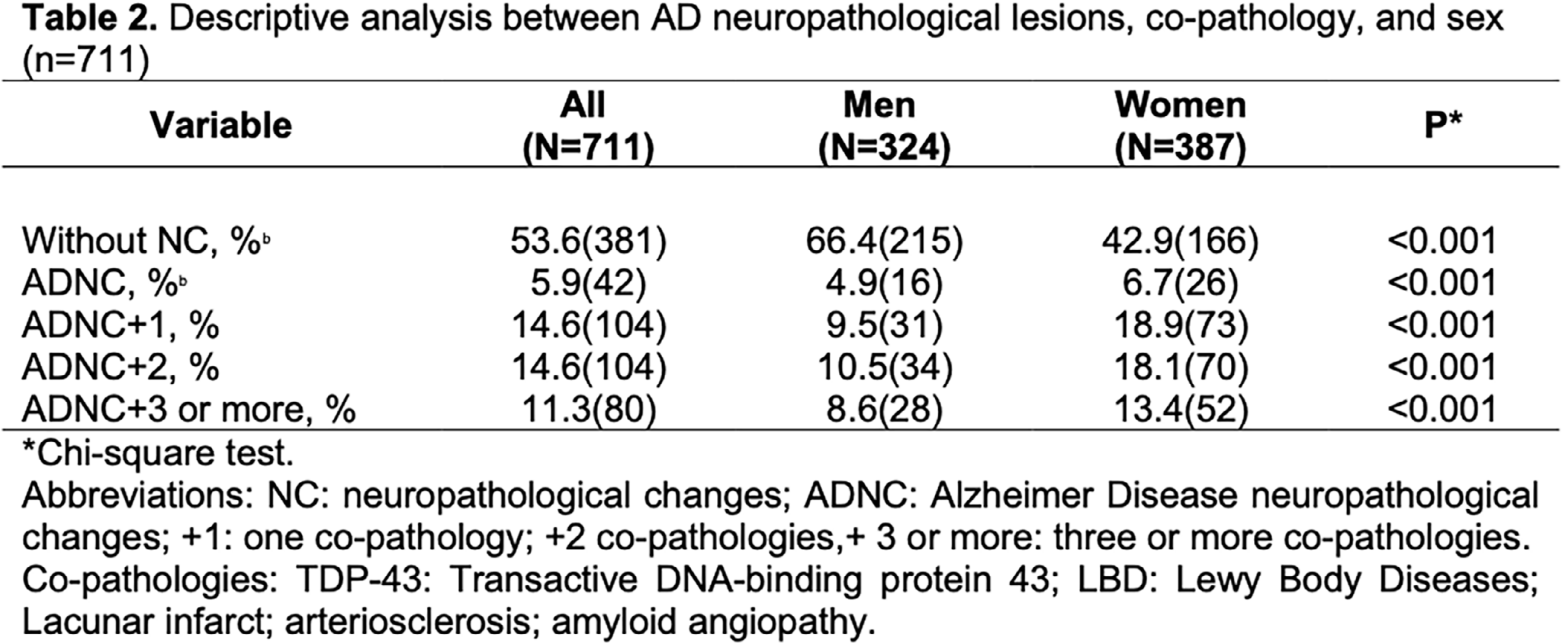# Sex differences mixed neuropathologies to Alzheimer's disease and cognitive abilities

**DOI:** 10.1002/alz70855_104882

**Published:** 2025-12-24

**Authors:** Karen L R Socher, Alberto Fernando Oliveira Justo, Roberta Diehl Rodriguez, Renata Elaine Paraizo Leite, Wilson Jacob‐Filho, Carlos Augusto Pasquallucci, Ricardo Nitrini, Lea T. Grinberg, Sonia Maria Dozzi Brucki, Claudia Kimie Suemoto

**Affiliations:** ^1^ Hospital das Clinicas ‐ FMUSP, São Paulo, Brazil; ^2^ Biobank for Aging Studies of the University of São Paulo, São Paulo, São Paulo, Brazil; ^3^ University of Sao Paulo Medical School, São Paulo, Brazil; ^4^ Cognitive and Behavioral Neurology Unit ‐ University of São Paulo, São Paulo, Brazil; ^5^ University of São Paulo Medical School, São Paulo, São Paulo, Brazil; ^6^ LIM44, Departamento de Radiologia e Oncologia, Faculdade de Medicina da Universidade de São Paulo, Sao Paulo, Brazil; ^7^ Physiopathology in Aging Laboratory (LIM‐22), University of Sao Paulo Medical School, São Paulo, São Paulo, Brazil; ^8^ Universidade de São Paulo ‐ USP, São Paulo, Brazil; ^9^ Brazilian Brain Bank of the Aging Brain Study Group; University of São Paulo, São Paulo, Brazil; ^10^ Biobank for aging studies of the University of São Paulo, São Paulo, Brazil; ^11^ University of São Paulo, São Paulo, Brazil; ^12^ Physiopathology in Aging Laboratory (LIM‐22), University of São Paulo Medical School, São Paulo, São Paulo, Brazil; ^13^ Biobank for Aging Studies of the University of São Paulo, Sao Paulo, Sao Paulo, Brazil; ^14^ Cognitive and Behavioural Neurology Unit, Hospital das Clínicas, University of São Paulo, São Paulo, São Paulo, Brazil; ^15^ Physiopathology in Aging Laboratory (LIM‐22), Department of Internal Medicine, University of São Paulo Medical School, São Paulo, São Paulo, Brazil

## Abstract

**Background:**

Sex differences have been investigated in Alzheimer's disease neuropathological changes (ADNC). However, few studies analyzed the occurrence of multiple co‐pathological changes. This study examined sex differences in AD neuropathology, co‐pathology, and cognitive abilities in a diverse sample.

**Method:**

Participants were from the Biobank for Aging Studies, a large population‐based autopsy study in Brazil. The neuropathological assessment includes AD pathology (neuritic plaques and neurofibrillary tangles), TDP‐43, Lewy body pathology, lacunar infarct, hyaline arteriolosclerosis, and cerebral amyloid angiopathy. Cognitive abilities were evaluated using the Clinical Dementia Rating Sum of Boxes (CDR‐SOB). We used ordinal logistic and linear regressions to investigate the associations of sex, education, and race with neuropathological and cognitive data adjusted for sociodemographic and clinical variables.

**Result:**

Among the 711 participants, 324 were men, and 387 were women; the mean age was 74.2±13.1 years old, and 67.4% were white. Women were older, less educated, and exhibited poor cognitive abilities compared to men in CDR‐SOB (β 5.8 CI=0.0‐15.0, *p* <0.001). Women showed a more significant frequency of AD pathology and co‐pathologies (*p* <0.001). The most frequent co‐pathologies were: Poorer cognitive abilities in women were associated with the number of co‐pathologies associated with ADNC, even when data was adjusted by age of death, education, race, hypertension, dyslipidemia, and diabetes.

**Conclusion:**

Women had poor cognitive abilities and higher association of ADNC and co‐pathologies than men.